# Epithelial-Mesenchymal-Transition-Like Circulating Tumor Cell-Associated White Blood Cell Clusters as a Prognostic Biomarker in HR-Positive/HER2-Negative Metastatic Breast Cancer

**DOI:** 10.3389/fonc.2021.602222

**Published:** 2021-06-02

**Authors:** Xiuwen Guan, Chunxiao Li, Yiqun Li, Jiani Wang, Zongbi Yi, Binliang Liu, Hongyan Chen, Jiasen Xu, Haili Qian, Binghe Xu, Fei Ma

**Affiliations:** ^1^ Department of Medical Oncology, National Cancer Center/National Clinical Research Center for Cancer/Cancer Hospital, Chinese Academy of Medical Sciences and Peking Union Medical College, Beijing, China; ^2^ State Key Laboratory of Molecular Oncology, National Cancer Center/Cancer Hospital, Chinese Academy of Medical Sciences and Peking Union Medical College, Beijing, China; ^3^ SurExam Bio-Tech, Guangzhou, China

**Keywords:** metastatic breast cancer, circulating tumor cells, white blood cell, epithelial-mesenchymal-transition, prognosis

## Abstract

**Background:**

Although positive Circulating tumor cells (CTCs) status has been validated as a prognostic marker in breast cancer, the interaction between immune cells and CTCs during the progress of Epithelial-mesenchymal-transition (EMT), and the clinical implications of CTC-associated white blood cell clusters (CTC-WBC clusters) for metastatic breast cancer are largely uncharacterized.

**Methods:**

We optimized a filter-based method combined with an RNA *in situ* hybridization technique according to the epithelial- and mesenchymal-markers to analyze EMT in CTC-WBC clusters. Serial peripheral blood samples from 135 patients with Hormone receptor (HR)-positive/HER2-negative metastatic breast cancer receiving first-line chemotherapy with docetaxel plus capecitabine were prospectively collected until disease progression from Nov 2013 to March 2019. Follow-up data collection was conducted until July 2020.

**Results:**

A total of 452 blood samples at all time-points were collected and analyzed. Median age of the cohort was 51.0 years (range, 27 to 73 years), and most of them (76.3%) had visceral metastases. Median progression-free survival (PFS) was 10.6 months (95% CI, 8.8 to 12.3 months). The presence of EMT-like CTC-WBC clusters was more frequently evident among patients with simultaneous bone and lymph node metastases (87.5% *vs* 36.2%, *P*=0.006), whereas no associations were observed between CTC-WBC clusters and other clinicopathologic characteristics before chemotherapy. The patients with EMT-like CTC-WBC clusters tended to show a significantly increased number of total CTC count (median,19.0 *vs* 5.0, *P*<0.001). The patients with at least one detectable EMT-like CTC-WBC cluster at baseline were characterized by significantly worse PFS, when compared to the patients with no EMT-like CTC-WBC clusters detected (7.0 *vs* 10.7 months, *P*=0.023), and those with five or more epithelial-based CTCs detected per 5mL of peripheral blood (7.0 *vs* 12.7 months, *P*=0.014). However, the total CTC-WBC clusters were not correlated with patients’ survival in the cohort (8.4 *vs* 10.6 months, *P*=0.561).

**Conclusions:**

Our data provide evidence that the emergence of CTC-WBC clusters underwent EMT before treatment is associated with significantly poorer PFS in HR-positive/HER2-negative metastatic breast cancer patients receiving docetaxel plus capecitabine, which may be used as a parameter to predict the clinical outcomes and a potential target for individualized therapy.

## Introduction

Circulating tumor cells (CTCs), potentially involved in the metastatic cascade (1), has been identified as a poor prognostic clinical factor in breast, lung, colorectal, and prostate cancer, which plays a key role in tumor cell dissemination for metastatic formation (2–6). Owing to the independent prognostic value of CTC count proved in multiple studies, the 8th edition of the American Joint Committee on Cancer (AJCC) cancer staging manual suggested CTCs as an adverse prognosis factor for patients with primary and metastatic breast cancer (7). However, carcinoma cells may undergo an epithelial-mesenchymal-transition (EMT) process and result in decreased expression of epithelial markers, which leads to false-negative findings by traditional epithelial marker-based CTC capturing methods (8).

The process of EMT is a key event in promoting stationary tumor cells to invade and migrate during metastatic cascade (6), which may also result in down-regulated expression of epithelial markers and an increase in mesenchymal proteins in CTCs. CTCs with the expression of mesenchymal markers, like TWIST1, SNAIL1, or vimentin were more likely to be identified in patients with advanced cancer compared to those with early-stage cancer, suggesting the potential role in metastatic dissemination and disease progression (8). Previous evidence in breast, lung, and colorectal cancer showed the presence of CTCs undergoing EMT was correlated with a higher risk of treatment failure and poor prognosis (9–13). Whereas, traditional epithelial marker-based CTC capturing methods, such as Cellsearch, may partially leak the detection of CTCs underwent EMT, thus it is necessary to optimize the enrichment and characterization system based on the expression of both epithelial- markers and mesenchymal-markers.

Moreover, CTCs may have direct interactions with immune cells during cancer dissemination, and correlate with the inflammatory state in the target organ of metastatic extravasation (14). Analyses of the interaction between CTCs and immune cells may provide reference for real-time therapy stratification and prognosis prediction. Although positive CTC status has been validated as a prognostic marker for recurrence-free survival, progression-free survival and overall survival in breast cancer (2, 3, 15), the interaction between immune cells and CTCs during the process of EMT, and the clinical implications of CTC-associated white blood cell clusters (CTC-WBC clusters) for metastatic breast cancer are largely uncharacterized. To address the technical challenges in a practical method and further investigate its clinical value, we optimized a filter-based method combined with an RNA *in situ* hybridization technique according to the epithelial- and mesenchymal-markers to analyze EMT in CTC-WBC clusters, and investigated the predictive value of different phenotypes of CTC-WBC clusters as a prognostic biomarker in a prospective study for Hormone receptor (HR)-positive/HER2-negative metastatic breast cancer.

## Methods

### Isolation and Classification of CTCs and CTC-WBC Clusters

We optimized the CanPatrol^®^ CTC enrichment technique for the detection of CTC and CTC-WBC clusters (16, 17). CTCs were isolated using a filter-based method combined with an RNA *in situ* hybridization method based on the branched DNA signal amplification technology according to the epithelial-markers EpCAM and CK8/18/19, the mesenchymal-markers vimentin and twist, and the leukocyte-marker CD45.

A total of 5 ml of patient peripheral blood sample was collected in a K2-EDTA tube and transferred to a sample preservative tube which contained lysing buffer *via* a tailored connection device. Erythrocytes were lysed by the red blood cell lysis buffer in 30 min at room temperature. The cell pellets were collected by centrifugation at 600g for 5 min and then PBS containing 4% formaldehyde was used to resuspend the remaining cells for 8 min. At last, CTCs were isolated in a filtration system by a calibrated membrane with 8μm pores filters which can separate small leukocytes from the large epithelial cells.

A multiplex mRNA *in situ* hybridization (ISH) assay was used to identify and classify CTCs. The assay was performed in a 24-well plate at 40°C for 3 hours and a set of probes (EMT markers) was utilized *in situ* hybridization. The epithelial-markers expressed on CTCs were detected by a multi-marker probe (such as EpCAM, CK8, CK18 and CK19), and the mesenchymal-markers expressed on CTC were detected by a multi-marker probe (such as Twist1 and Vimentin). The CD45 probe was used as the leukocyte-marker. Finally, the cells were stained with DAPI for 10 min and analyzed with an automated imaging fluorescence microscope. The red and green fluorescent signals represent epithelial and mesenchymal markers expression, respectively. A bright white fluorescent signal represents CD45 expression. As **Figure 2A** depicted, CTC-WBC cluster was defined as CTC karyotype connected to typical WBC karyotype cells, on which CD45 fluorescence signal points expressed.

### Patient Cohort Selection

A total of 135 patients with HR-positive/HER2-negative metastatic breast cancer receiving first-line chemotherapy with docetaxel plus capecitabine were prospectively enrolled in the observation cohort to investigate the clinical implications of different phenotypes of CTC-WBC clusters. The patients were recruited after an agreement from the local Ethical committee between Nov 2013 to March 2019 from 32 clinical centers in China. Key eligibility included (1) confirmed histologic/cytologic diagnosis of HR-positive/HER2-negative metastatic breast cancer, (2) previously untreated with first-line chemotherapy, (3) age≥18, (4) Eastern Cooperative Oncology Group performance status < 2, (5) with measurable lesions defined by revised Response Evaluation Criteria in Solid Tumors guidelines version 1.1 (RECIST 1.1), and (6) adequate hematologic, hepatic and renal function.

The clinicopathological characteristics, including age, estrogen receptor (ER) status, progesterone receptor (PR) status, disease free survival (DFS), position of metastatic sites, and previous endocrinotherapy after confirmed tumor relapse were collected before enrollment. Enrolled patients received first-line chemotherapy of docetaxel plus capecitabine for a maximum of 6 cycles or until disease progression, intolerable adverse events, or patient withdrawal occurred, and those with stable disease or a partial or complete response after initial chemotherapy received the maintenance chemotherapy of capecitabine. Blood samples were collected from the baseline and dynamically tracked every 6 weeks during the treatment until disease progression. Follow-up data collection was conducted until July 2020.

### Statistical Analyses

The associations between the distribution of different subtypes of CTCs and the heterogeneity of CTC-WBC clusters were analyzed by Mann-Whitney U test. *χ* ^2^ test or Fisher’s exact test were used to compare the distribution of clinicopathological characteristics between different groups and described in percentages of categorical variables. Kaplan-Meier analysis was performed to investigate the prognostic value of CTC-WBC clusters and CTC-WBC clusters under EMT. Multivariate hazard ratios for PFS were estimated with Cox proportional hazards regression analysis. The statistical analyses were performed using SPSS software, version 23.0 (SPSS Inc., Chicago, IL, USA). All *P* values were 2-sided and considered to be statistically significant when less than 0.05.

## Results

### Clinicopathologic Characteristics of the Enrolled Cohort

A total of 135 female patients with HR-positive/HER2-negative metastatic breast cancer were recruited in the study for CTC assessment. The median age of the cohort was 51.0 years (range, 27 to 73 years). Among them, 103 (76.3%) of the patients had visceral metastasis. 20 (14.8%) of the patients were *de novo* Stage IV breast cancer and 36 (26.7%) of them were found relapsed or metastasis within 24 months. 32 (23.7%) of the Luminal-like patients received endocrinotherapy after confirmed tumor relapse.

In the study, disease progression was observed in totally 108 patients and the median follow-up was 36.0 months (95% CI, 27.8 to 44.3 months). Median progression-free survival of the cohort was 10.6 months (95% CI, 8.8 to 12.3 months).

### Association Between Different Subtypes of CTCs and the Heterogeneity of CTC-WBC Clusters

**Figure 1:**
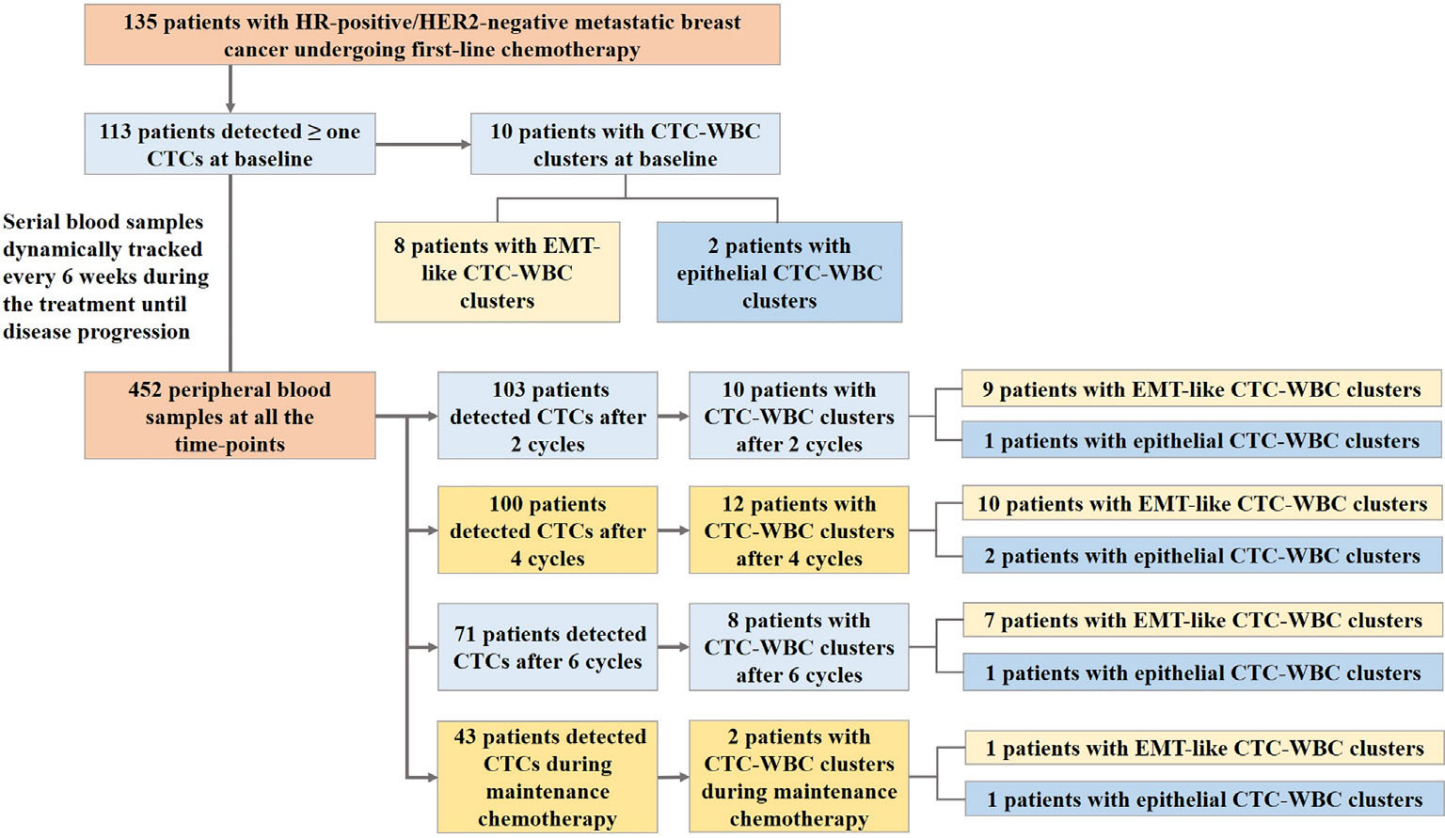
Flow diagram of the study.

Serial peripheral blood samples from the enrolled 135 patients were prospectively collected during the first-line chemotherapy of docetaxel plus capecitabine, with the median of 3 serial samples collected from each patient. A total of 452 blood samples at all time-points were collected and analyzed (**Figure 1**). In the whole cohort,381 (84.3%) of the blood samples at all time-points were detected with more than one CTCs and 42 (9.3%) were detected with CTC-WBC clusters. In the study, totally 53 CTC-WBC clusters were captured, including 10 epithelial CTC-WBC clusters (with epithelial biomarkers detected on CTCs), 39 biphenotypic epithelial/mesenchymal CTC-WBC clusters (with both epithelial and mesenchymal biomarkers detected on CTCs) and 4 mesenchymal CTC-WBC clusters (with mesenchymal biomarkers detected on CTCs).

As **Table 1** showed, the patients with CTC-WBC clusters detected were simultaneously observed in significantly increased enumeration of each subtypes of CTC count, especially in E/M-CTCs and M-CTCs. Among the patients with more than 5 CTCs, the percentage of the emergency of CTC-WBC clusters in the cohorts was eight times increased than those with CTC<5 (14.4% *vs* 1.6%, χ^2^ = 21.1, *P*<0.001). Moreover, in the patients with more than 20 CTCs, the presence of CTC-WBC clusters was more than three times frequency than those CTC<20 (25.6% *vs* 5.9%, χ^2^ = 29.9, *P*<0.001).

**Table 1:** The correlation between different subtypes of CTCs and the presence of CTC-WBC clusters as well as EMT-like CTC-WBC clusters.

	CTC-WBC clusters				EMT-like CTC-WBC clusters			
	with	without	Z score	*P* value	with	without	Z score	*P* value
Total CTCs^†^	19.0 (8.0,28.0)	5.0 (2.0,13.0)	-5.975	<0.001	19.0 (8.0,28.0)	5.0 (2.0,13.0)	-5.432	<0.001
E-CTCs^†^	2.0 (0,6.25)	0 (0,2.0)	-3.099	0.002	1.0 (0,5.0)	1.0 (0,2.5)	-1.649	0.099
E/M-CTCs^†^	8.0 (3.0,24.0)	2 (0,7.0)	-5.658	<0.001	9.0 (4.0,24.0)	2.0 (0,6.5)	-5.702	<0.001
M-CTCs^†^	2.0 (0,9.25)	1 (0,2.0)	-3.299	0.001	3.0 (0,10.0)	1.0 (0,2.0)	-3.387	0.001
EMT-CTCs^†^	13.5 (6.0,27.0)	4 (1.0,10.0)	-5.553	<0.001	16.0 (6.0,28.0)	4.0 (1.0,10.0)	-5.628	<0.001
Proportion of EMT-CTCs	90.8%	82.0%	-1.904	0.057	94.7%	80.0%	-2.773	0.006

^†^median (interquartile range, per 5mL).

E-CTCs, epithelial CTCs; E/M-CTCs, biphenotypic epithelial/mesenchymal CTCs; M-CTCs, mesenchymal CTCs; EMT-CTCs, CTCs underwent EMT, including E/M-CTCs and M-CTCs.

**Figure 2:**
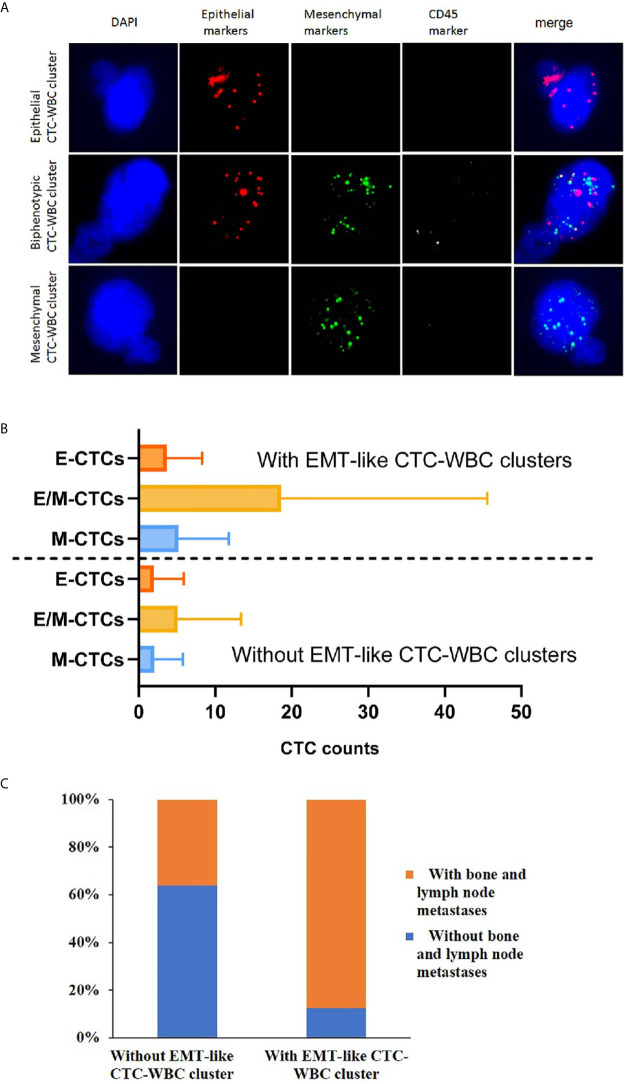
Association of the detection of EMT-like CTC-WBC clusters with different subtypes of CTCs and the clinicopathological characteristics. **(A)** Representative images of CTC-WBC clusters classified by categorical markers. **(B)** The distribution of different CTC subtypes in the patients with EMT-like CTC-WBC clusters and those patients without. **(C)** Association between the detection of EMT-like CTC-WBC clusters and metastatic sites.

As for the patients detected with CTC-WBC clusters underwent mesenchymal transformation (EMT-like CTC-WBC clusters, including biphenotypic epithelial/mesenchymal CTC-WBC clusters and mesenchymal CTC-WBC clusters), a significantly increased number of total CTCs, E/M-CTCs and M-CTCs were simultaneously observed when compared to those without EMT-like CTC-WBC clusters detected (*P*<0.05, **Figure 2B** and **Table 1**). Besides that, significantly increased proportion of EMT-CTCs (CTCs underwent EMT, including E/M-CTCs and M-CTCs) in total CTC count was observed in the patients with EMT-like CTC-WBC clusters captured (median, 94.7% *vs* 80.0%, *P*=0.006). Similarly, in patients with more than 5 CTCs and those with more than 20 CTCs, the frequency of presence of EMT-like CTC-WBC clusters was significantly higher than those with low CTC counts (For CTCs≥5 *vs* CTCs<5: 11.9% *vs* 1.6%, χ2 = 15.8, *P*<0.001; For CTCs≥20 *vs* CTCs <20: 21.8% *vs* 4.8%, χ2 = 26.1, *P*<0.001).

### Association Between the Emergence of CTC-WBC Clusters and Clinicopathological Features

Before the start of first-line chemotherapy with docetaxel plus capecitabine at baseline, 113 (83.7%) of the 135 patients had more than one detectable CTCs with a median number of 6 CTCs per 5 ml of peripheral blood sample (range, 1 to 151 CTCs/5mL). Meanwhile, CTC-WBC clusters were detectable in 10 patients at baseline, of whom 8 patients had EMT-like CTC-WBC clusters.

When comparing the clinicopathological characteristics between the cohort with EMT-like CTC-WBC cluster detected at baseline and those without, the presence of CTC-WBC clusters had no association with age, menstrual status, ER status, PR status, DFS, and previous endocrinotherapy after confirmed relapse or metastasis (**Table 2**). Concerning the association with different metastatic sites of the enrolled patients, the presence of EMT-like CTC-WBC clusters at baseline was more frequently evident among patients with simultaneous bone metastases and lymph node metastases (87.5% *vs* 36.2%, *P*=0.006, **Figure 2C**), whereas no significant correlation was observed between the detection of EMT-like CTC-WBC clusters and other metastatic sites (**Table 2**).

**Table 2:** The association between the presence of EMT-like CTC-WBC clusters and the clinicopathological characteristics of 135 enrolled patients with HR-positive/HER2-negative metastatic breast cancer undergoing docetaxel plus capecitabine as first-line chemotherapy.

	Without EMT-like CTC-WBC clusters at baseline	With EMT-like CTC-WBC clusters at baseline	*P* value
Age			0.353
<60	101 (79.5%)	8 (100.0%)	
≥60	26 (20.5%)	0	
Menstrual status			0.138
Premenopausal	54 (42.5%)	6 (75.0%)	
Menopause	73 (57.5%)	2 (25.0%)	
ER status			0.267
Positive	123 (96.9%)	7 (87.5%)	
Negative	4 (3.1%)	1 (12.5%)	
PR status			1.000
Positive	116 (91.3%)	8(100.0%)	
Negative	11 (8.7%)	0	
Disease-free survival			0.439
<24 months	33 (26.0%)	3 (37.5%)	
≥24 months	94 (74.0%)	5 (62.5%)	
Position of metastatic site			0.090
Non-visceral	28 (22.0%)	4 (50.0%)	
Visceral	99 (78.0%)	4 (50.0%)	
Liver metastases			0.726
Without	68 (53.5%)	5 (62.5%)	
With	59 (46.5%)	3 (37.5%)	
Lung metastases			0.154
Without	59 (46.5%)	6 (75.0%)	
With	68 (53.5%)	2 (25.0%)	
Bone metastases			0.144
Without	53 (41.7%)	1 (12.5%)	
With	74 (58.3%)	7 (87.5%)	
Lymph node metastases			0.268
Without	44 (34.6%)	1 (12.5%)	
With	83 (65.4%)	7 (87.5%)	
Simultaneous bone and lymph node metastases			0.006
Without	81 (63.8%)	1 (12.5%)	
With	46 (36.2%)	7 (87.5%)	
Previous endocrinotherapy (after confirmed relapse)			0.849
None	97 (76.4%)	6 (75.0%)	
1st-line	26 (20.5%)	2 (25.0%)	
2nd-line or more	4 (3.1%)	0	

### Association Between the Heterogeneity of CTC-WBC Clusters and Progression-Free Survival

**Figure 3:**
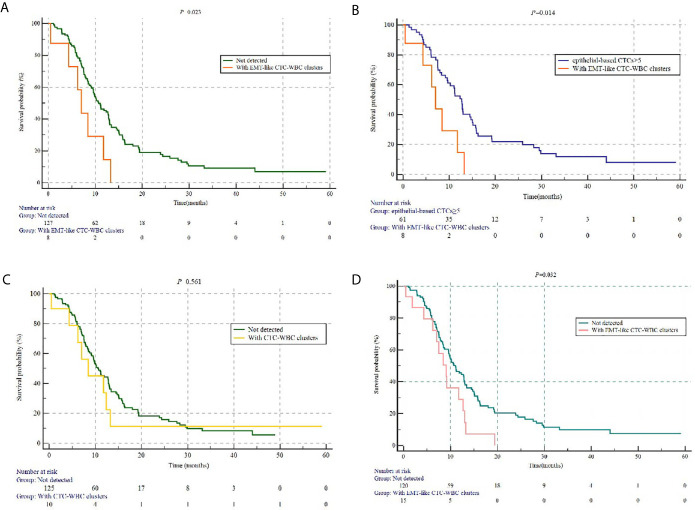
The prognostic value of different subtypes of CTC-WBC clusters. **(A)** Kaplan-Meier analysis-based estimation of PFS probabilities of comparing the patients with at least one EMT-like CTC-WBC clusters to those without. **(B)** Kaplan-Meier analysis-based estimation of PFS probabilities of comparing the patients with at least one EMT-like CTC-WBC clusters to those to five or more epithelial-based CTCs. **(C)** Kaplan-Meier analysis-based estimation of PFS probabilities of comparing the patients with at least one CTC-WBC clusters to those without. **(D)** Kaplan-Meier analysis-based estimation of PFS probabilities of comparing the patients with at least one EMT-like CTC-WBC clusters within 6 weeks after the start of chemotherapy to those without.

In this relatively homogeneous HR-positive/HER2-negative metastatic breast cancer cohort, no significant difference was observed in the progression-free survival (PFS) between the patients with five or more CTCs and those with fewer than five CTCs per 5 mL of peripheral blood at baseline before initiation of first-line chemotherapy (9.5 months *vs* 11.2 months, *P*=0.387). Nevertheless, the patients with at least one detectable EMT-like CTC-WBC clusters at baseline were characterized by significantly worse PFS, when compared to the patients with no EMT-like CTC-WBC clusters detected (7.0 months *vs* 10.7 months, *P*=0.023; **Figure 3A**), as well as the patients with at least one CTC detected per 5 ml of peripheral blood (7.0 months *vs* 11.2 months, *P*=0.019), and those with five or more epithelial-based CTCs (including E-CTCs and E/M-CTCs, parallel to isolation of CTCs by epithelial markers; 7.0 months *vs* 12.7 months, *P*=0.014; **Figure 3B**). Moreover, multivariate analysis showed the presence of EMT-like CTC-WBC clusters remained the only significantly independently predictive factors associated with PFS, with the adjusted HR of 2.415 (95% CI:1.046, 5.574, *P*=0.039, detail data of other clinical indexes in the multivariate analysis showed in **Supplementary Table 1**). However, the total CTC-WBC clusters and epithelial-based CTC-WBC clusters (including epithelial CTC-WBC clusters and biphenotypic epithelial/mesenchymal CTC-WBC clusters) were not correlated with patients’ survival in the cohort, respectively (8.4 months *vs* 10.6 months, *P*=0.561, **Figure 3C**; 8.4 months *vs* 10.7 months, *P*=0.511, **Table 3**).

**Table 3:** Detail description of Kaplan-Meier analysis-based estimation of Progression-free survival for different subtypes of CTCs and CTC-WBC clusters at baseline.

	different subtypes of CTCs and CTC-WBC clusters at baseline	No. of patients	No. of patients progressed	Median Progression-free survival (months)	*P* value
Index 1	Total CTC counts≥5	84	71	11.2	0.387
	Total CTC counts<5	51	37	9.5	
Index 2	epithelial-based CTCs≥5	68	57	11.8	0.362
	epithelial-based CTCs<5	67	51	9.6	
Index 3	EMT-like CTC-WBC clusters	8	7	7.0	0.023
	no EMT-like CTC-WBC clusters	127	101	10.7	
Index 4	EMT-like CTC-WBC clusters	8	7	7.0	0.019
	Total CTC counts≥1	105	87	11.2	
Index 5	EMT-like CTC-WBC clusters	8	7	7.0	0.013
	Total CTC counts≥5	77	65	12.3	
Index 6	EMT-like CTC-WBC clusters	8	7	7.0	0.014
	epithelial-based CTCs≥5	61	51	12.7	
Index 7	CTC-WBC clusters	10	8	8.4	0.561
	no CTC-WBC clusters	125	100	10.6	
Index 8	epithelial-based CTC-WBC clusters	9	7	8.4	0.511
	no epithelial-based CTC-WBC clusters	126	101	10.7	

As for patients detected with EMT-like CTC-WBC clusters within 6 weeks after the initiation of chemotherapy (within the first follow-up visit), statistically significantly shorter PFS was observed when compared with the patients without (9.1 months *vs* 11.0 months, *P*=0.032, **Figure 3D**). However, no parallel results can be summarized from the patients with EMT-like CTC-WBC clusters detected after 6 weeks during the first-line treatment.

## Discussions

Despite the prognostic value of CTCs has been discussed in many attempts, the clinical significance of the interaction between immune cells and CTCs during the process of Epithelial-mesenchymal-transition are largely uncharacterized. We optimized a filter-based method combined with an RNA *in situ* hybridization technique according to both the epithelial- and mesenchymal-markers, and investigated the predictive value of different phenotypes of CTC-WBC clusters as a prognostic biomarker. Our study revealed the prognostic value of CTC-WBC cluster underwent mesenchymal transformation in a prospective cohort composed of relatively homogeneous participants who were all HR-positive/HER2-negative metastatic breast cancer underwent first-line chemotherapy of docetaxel plus capecitabine, on which epithelial-to-mesenchymal transition was correlated with drug resistance (18, 19).

Several studies illuminated CTC clusters were more likely to be present in patients with high enumeration of CTCs (20, 21). Similar association was found between CTC-WBC clusters and single CTCs in our present study, that is, the frequency of presence of CTC-WBC clusters increased in patients with high enumeration of CTC count, especially in those with high numbers of M-CTCs and E/M-CTCs. Owing to the fact bone marrow was a common homing organ for metastatic tumor cells (22), the detection of EMT-like CTC-WBC clusters at baseline was associated with simultaneous bone metastases and lymph node metastases in the present study, suggesting the correlation between tumor cells and immune cells during tumor dissemination. Increased metastatic propensity of CTC clusters in mouse models and adverse prognosis in breast cancer patients with abundant CTC clusters, suggested the role of CTC clusters as critical mediators of cancer metastasis (23). However, the correlation between CTC clusters and immune cells during cancer dissemination was unclear.

CTCs in the peripheral blood left the immunosuppressive microenvironment of the primary tumor and might be vulnerable to immune surveillance, which required immune−escape mechanisms if they were to form metastases, including alterations in the expression of MHC molecules, NK-cell ligands, FAS, FAS ligand, and immune-checkpoint molecules, such as CD47 and programmed cell death 1 ligand 1 (PD−L1) (14). Analyses of the interaction between CTCs and immune cells may provide reference for real-time therapy stratification and prognosis prediction. In a cohort of 106 patients with advanced non-small-cell lung cancer receiving first-line cisplatin-based chemotherapy, Ilié M et al. (24)found a trend for longer PFS and OS was observed in those with PD-L1 expression in CTCs or circulating WBCs. A recent study investigated the association between WBCs and CTCs during blood-borne dissemination by single-cell RNA sequencing and revealed that neutrophils directly interact with CTCs which drives cell cycle progression in circulation and accelerates the metastatic potential of CTCs (25). Szczerba BM et al. (25) observed that the patients of invasive breast cancer in whom at least one CTC-neutrophil cluster were detected per 7.5 ml of peripheral blood were correlated with significantly worse PFS compared to patients with five or more CTCs in 7.5 ml of peripheral blood. However, the study of Szczerba BM et al. (25) focused on the mechanism of interaction between CTCs and WBCs in breast cancer patients and mouse models, while the human samples involved in the study were very heterogeneous because of the recruitment from merely 34 patients with detectable CTCs of different tumor subtypes, TNM stage and diverse treatment. Thus, the statistical power of heterogenous group may be influenced by confounding factors. Moreover, the study did not disclose the clinical implications of CTC-WBC clusters during the progress of epithelial-mesenchymal transition.

However, when carcinoma cells are undergoing EMT process, reduced expression of epithelial markers and upregulated expression of mesenchymal markers on CTCs may result in false-negative findings in the cell surface epithelial marker-based CTC capturing methods (8). Therefore, the identification of additional upregulated mesenchymal markers on CTCs during EMT may lessen the leak detection of CTCs. Previous studies by Yu M et al. (11) and our research group (26) suggested that in patients with metastatic breast cancer, the fluctuation of proportion of CTCs underwent mesenchymal transformation may be more appropriate for predicting prognosis and evaluating therapeutic resistance compared to total CTC enumeration. Whereas, the clinical implications of EMT marker composition in CTC-WBC clusters were uncharacterized before.

In the present study, the patients with at least one EMT-like CTC-WBC clusters before treatment showed significantly shorter PFS compared to those with five or more epithelial-based CTCs. However, epithelial-based CTC-WBC clusters, including epithelial CTC-WBC clusters and biphenotypic epithelial/mesenchymal CTC-WBC clusters, did not reach the statistical power to predict the disease outcome in our study, owing to the lack of mesenchymal composition detected. In this prospective cohort composed of relatively homogeneous participants who were all HR-positive/HER2-negative breast cancer patients receiving first-line chemotherapy of docetaxel plus capecitabine, epithelial-to-mesenchymal transition was positively correlated with drug resistance (18, 19), which may lead to the failure in the prognostic prediction of simple total CTC count and epithelial-based CTC-WBC clusters. Even though the occurrence of EMT-like CTC-WBC clusters seemed to be lower than CTCs, it may be a more accurate marker to differentiate the special population who were more likely resistant to the current treatment, for whom the combination of targeting residual EMT-driven cancer cells in addition to conventional therapy may decrease metastasis formation and drug resistance (27). Meanwhile, blocking the process of epithelial-to-mesenchymal transition and reducing EMT-like CTC-WBC clusters may provide a therapeutic target to control metastatic spread and increase the efficacy of anticancer treatments. To our knowledge, the study is the first attempt to characterize the prognostic value of EMT-like CTC-WBC clusters in a homogeneous cohort, which provide evidence for clinical transformation. However, limited sample size influenced the statistical power to draw firm conclusions, and further large-scale external validation containing data of overall survival is warranted. Besides that, the expression of some EMT-related genes, such as ESRP1 and RBFOX2 (28), which correlated with cancer progression, may also be explored in the EMT-like CTC-WBC clusters in further study to enrich the prognostic model.

In summary, our investigation suggested that the presence of EMT-like CTC-WBC clusters before treatment was associated with significantly poorer PFS in HR-positive/HER2-negative metastatic breast cancer patients receiving first-line chemotherapy of docetaxel plus capecitabine, raising a concern on the optimized choice of first-line treatment for these patients. The results suggested that taking both the cancer cells and immune cells into consideration in the liquid-microenvironment biopsy, EMT-like CTC-WBC clusters may serve as a potential biomarker for prognosis prediction and a rationale target for individualized therapy in HR-positive/HER2-negative metastatic breast cancer patients.

## Data Availability Statement

The raw data supporting the conclusions of this article will be made available by the authors, without undue reservation.

## Ethics Statement

The studies involving human participants were reviewed and approved by the Independent Ethics Committee of Cancer Hospital, Chinese Academy of Medical Sciences and National GCP Center for Anticancer Drugs. The patients/participants provided their written informed consent to participate in this study. Written informed consent was obtained from the individual(s) for the publication of any potentially identifiable images or data included in this article.

## Author Contributions

FM, BX, and HQ conceived the study. XG, CL, YL, JW, ZY, BL, HC, and JX collected and analyzed the data. XG wrote the whole manuscript. All authors contributed to the article and approved the submitted version.

## Funding

The study was supported with National Nature Science Foundation of China (81874122), CAMS Initiative for Innovative Medicine (2017-I2M-3-004), PUMC Innovation Fund for Doctors (2018-1002-02-24) and China Postdoctoral Science Foundation(2020M680455).

## Conflict of Interest

JX was employed by SurExam Bio-Tech.

The remaining authors declare that the research was conducted in the absence of any commercial or financial relationships that could be construed as a potential conflict of interest.

## Supplementary Material

Supplementary Table 1 Multivariate analysis of clinicopathological characteristics and the detection of CTCs and EMT-like CTC-WBC clusters for predicting the progression-free survival in HR-positive/HER2-negative metastatic breast cancer patients.
