# Supplementary material for: Epithelial-Mesenchymal-Transition-Like Circulating Tumor Cell-Associated White Blood Cell Clusters as a Prognostic Biomarker in HR-Positive/HER2-Negative Metastatic Breast Cancer
Source: Front Oncol. 2021 Jun 2;11:602222. doi: 10.3389/fonc.2021.602222 (PMC8208036; doi:10.3389/fonc.2021.602222)
Supplement: Supplementary Table 1 — Multivariate analysis of clinicopathological characteristics and the detection of CTCs and EMT-like CTC-WBC clusters for predicting the progression-free survival in HR-positive/HER2-negative metastatic breast cancer patients. [file Table_1.docx]

Supplementary Table 1. Multivariate analysis of  clinicopathological characteristics and the detection of EMT-like CTC-WBC clusters for predicting the progression-free survival in HR-positive/HER2-negative metastatic breast cancer patients

| Variable | Reference groups | HR | 95.0% CI | *P* value |
| --- | --- | --- | --- | --- |
| EMT-like CTC-WBC clusters at baseline | Positive vs Negative | 2.415 | 1.046,5.574 | 0.039 |
| Age | <60 vs ≥60 | 0.943 | 0.555,1.601 | 0.827 |
| ER status | Positive vs Negative | 0.933 | 0.368,2.366 | 0.884 |
| PR status | Positive vs Negative | 0.948 | 0.486,1.850 | 0.875 |
| Visceral metastases | Without vs With | 0.982 | 0.618,1.561 | 0.940 |
| Simultaneous bone and lymph node metastases | Without vs With | 0.992 | 0.661,1.490 | 0. 970 |
| Previous endocrinotherapy | Without vs With | 0.705 | 0.437,1.138 | 0.152 |
